# House Physician or House Surgeon?

**Published:** 1907-07-27

**Authors:** 


### RESIDENT MEDICAL OFFICERS' DEPARTMENT.

[Contributions to this Column are invited, and, if accepted, will be paid for.]

### HOUSE PHYSICIAN OR HOUSE SURGEON?

A QUESTION which presents itself to many newly-qualified men, when they think of applying for house appointments, is this: "Which will be the more useful post to me in after life—house surgeon or house physician?" The answer depends upon a number of things. In the first place the means and ambitions of the intending applicant himself have to be considered: what are his plans for the future? Secondly, the kind of institution he wishes to serve in—large or small, general or special. Thirdly, his choice must be to some extent guided by the vacancies that present themselves, and the competition that he is likely to meet with in his application. Fourthly, he must make up his mind whether he wishes to take a series of hospital appointments, for this should be an important factor in his choice.

### CONSIDERATIONS.

Having decided, so far as he is able, upon the line of practice which he means to adopt in after life, the aspirant to the post of house officer should carefully weigh the other considerations that we have mentioned, before he canvasses his teachers for testimonials and personal recommendations, and before he sends in his application. Should he aspire to become a consulting surgeon, or a specialist in one of the various branches of surgery, the newly-qualified man will of course select the post of house surgeon for the first step in his triumphant career, and the larger the hospital in which he holds this appointment the better for his ultimate chances of success. If his merits are proportionate to his ambitions he should have no difficulty in becoming house surgeon at his own hospital as soon as he chooses to apply for the post. The same holds true of the office of house physician in the case of him who aspires to medical honours.

For the man who proposes to enter one of His Majesty's Services there is more difficulty in choosing between the rival attractions of a house surgeonship and a house physicianship. The experience gained by holding the latter appointment is no doubt of more value in time of peace, for the bulk of practice in the Services is medical; but a reputation for skill in surgery is, perhaps, a greater advantage at any time for purposes of promotion, and familiarity with operative surgery is, of course, an enormous asset on active service. On the whole, therefore, it is advisable for him to take the post of house surgeon if the choice lies with him.

The man with a strong taste for the more exact sciences, who proposes to return to the delights of pure research soon after he has taken a medical degree, often decides to hold a house appointment, by way of adding a leaf to his crown of laurels, or perhaps of taking a final survey of the despised art of healing. To him, after mature consideration, we would recommend a house physicianship, as offering more leisure for research and study, and better opportunities for theorisation and scientific conjecture.

### THE GENERAL PRACTITIONER'S CHOICE.

We now come to the man who, of his own choice or through force of circumstances, is destined to enter the ranks of general practice. Since at least 80 per cent. of all his future work will be medical, we would advise him (if he cannot spare the time to take more than one hospital appointment) to apply for the post of house physician to the largest general hospital at which he has a reasonable hope of being appointed. The casualty practice there will be of infinite value to him in the ordinary run of his subsequent work; while the in-patient practice will train him in habits of careful and exhaustive investigation at the bedside, which he will be able to apply to those cases in after life which are worthy of something more than perfunctory examination and faint-hearted diagnosis.

If he can possibly manage to take afterwards a short surgical appointment, say, of six months, at some hospital whose visiting surgeons are of good repute, and another short appointment as resident obstetric officer, where there are opportunities for practising both midwifery and gynæcology, he will have reaped an experience of hospital methods sufficient to place him in the very front rank of general practitioners.

### PROVINCIAL APPOINTMENTS.

All, however, are not so fortunate as this: many have not the means or the ability or the address requisite for securing either a resident post for a year at a great metropolitan hospital, or a varied sequence of house appointments at different institutions. To them it is usually possible to secure house surgeonships at small provincial or suburban hospitals. Some of these are indeed sinecures, and for that reason to be avoided; but there are also many such posts which, when filled by energetic men, provide even in six months a large experience of every branch of medical and surgical practice.

There is one famous London hospital in which we understand the full term of junior staff appointment is 18 months: six months on probation as general clinical assistant, six months as resident house surgeon, and the last six months as resident house physician. This seems to be a logical sequence, affording an extended hospital experience of much variety. Our remarks on choosing a resident post do not, therefore, apply directly to this hospital, but more particularly to those in which, for the best of reasons, it is not permitted to the same man to hold in succession the offices both of house surgeon and of house physician.

